# Mitochondrial dysfunction and consequences in calpain-3-deficient muscle

**DOI:** 10.1186/s13395-020-00254-1

**Published:** 2020-12-11

**Authors:** Vanessa E. Jahnke, Jennifer M. Peterson, Jack H. Van Der Meulen, Jessica Boehler, Kitipong Uaesoontrachoon, Helen K. Johnston, Aurelia Defour, Aditi Phadke, Qing Yu, Jyoti K. Jaiswal, Kanneboyina Nagaraju

**Affiliations:** 1 Center for Genetic Medicine Research, Children’s National Research Institute, Children’s National Hospital, Washington, D.C., USA; 2 School of Exercise and Rehabilitation Sciences, The University of Toledo, Toledo, OH USA; 3 Department of Genomics and Precision Medicine, George Washington University School of Medicine and Health Sciences, Washington, D.C., USA; 4 School of Pharmacy and Pharmaceutical Sciences, SUNY Binghamton University, PO Box 6000, Binghamton, NY 13902 USA

**Keywords:** LGMD2A, Mitochondria, Calpain-3 deficiency, Muscle membrane repair

## Abstract

**Background:**

Nonsense or loss-of-function mutations in the non-lysosomal cysteine protease calpain-3 result in limb-girdle muscular dystrophy type 2A (LGMD2A). While calpain-3 is implicated in muscle cell differentiation, sarcomere formation, and muscle cytoskeletal remodeling, the physiological basis for LGMD2A has remained elusive.

**Methods:**

Cell growth, gene expression profiling, and mitochondrial content and function were analyzed using muscle and muscle cell cultures established from healthy and calpain-3-deficient mice. Calpain-3-deficient mice were also treated with PPAR-delta agonist (GW501516) to assess mitochondrial function and membrane repair. The unpaired *t* test was used to assess the significance of the differences observed between the two groups or treatments. ANOVAs were used to assess significance over time.

**Results:**

We find that calpain-3 deficiency causes mitochondrial dysfunction in the muscles and myoblasts. Calpain-3-deficient myoblasts showed increased proliferation, and their gene expression profile showed aberrant mitochondrial biogenesis. Myotube gene expression analysis further revealed altered lipid metabolism in calpain-3-deficient muscle. Mitochondrial defects were validated in vitro and in vivo. We used GW501516 to improve mitochondrial biogenesis in vivo in 7-month-old calpain-3-deficient mice. This treatment improved satellite cell activity as indicated by increased MyoD and Pax7 mRNA expression. It also decreased muscle fatigability and reduced serum creatine kinase levels. The decreased mitochondrial function also impaired sarcolemmal repair in the calpain-3-deficient skeletal muscle. Improving mitochondrial activity by acute pyruvate treatment improved sarcolemmal repair.

**Conclusion:**

Our results provide evidence that calpain-3 deficiency in the skeletal muscle is associated with poor mitochondrial biogenesis and function resulting in poor sarcolemmal repair. Addressing this deficit by drugs that improve mitochondrial activity offers new therapeutic avenues for LGMD2A.

## Background

Calpains are non-lysosomal, Ca^2+^-dependent, cysteine proteases [1]. In addition to the two ubiquitous calpain isoforms CAPN1 and CAPN2, the skeletal muscles also express CAPN3 protein also known as p93 or calpain-3 [2]. Although this 94-kDa Ca^2+^-dependent cysteine protease is known to localize to sarcomeres through binding the giant sarcomeric protein titin (also referred to as connectin) [3], its physiological functions are yet to be fully elucidated. CAPN3 has been implicated in muscle repair and maintenance [4], myogenesis [5], NF-κB signaling, and apoptosis [6, 7]. Loss-of-function mutations in the *CAPN3* gene lead to the autosomal recessive form of limb-girdle muscular dystrophy (LGMD) type 2A (LGMD2A).

LGMD2A is one of the most frequent subtypes of autosomal recessive muscular dystrophy, accounting for up to 30% of all recessive LGMD cases [8]. Disease onset usually occurs in the second decade of life but can vary from age 2.5 to 50 years [9–11]. Progression of the disease is slow, leading to loss of ambulation during adulthood and a near-normal life expectancy [12]. Histopathological features of the muscle include necrosis, regeneration, and variation in muscle fiber diameter [13–15]. More than 440 different pathogenic mutations of the *CAPN3* gene have been reported in LGMD2A patients [4]. These mutations are distributed along the entire length of the gene product and include missense, nonsense, frame-shift, and deletion mutations [15–18].

Three independent mouse models have been used to study the biology of CAPN3 and its role in diseases [6, 19, 20]. Findings from all three mouse models highlight the importance of CAPN3 in skeletal muscle biology, but the precise mechanism by which *CAPN3* mutations lead to the LGMD2A phenotype has remained unclear. Electron microscopic examination of LGMD2A patient biopsies has implicated mitochondrial abnormalities in the skeletal muscle, providing insights into one possible mechanism in the disease process [21]. Mitochondria play important roles in regulating muscle repair and muscle mass maintenance [22–29]. The physiological importance of mitochondrial activity has for many decades been associated with ATP synthesis and the maintenance of an energetic steady state in the cell. However, numerous studies have demonstrated that mitochondria are not only a locus of energy synthesis but also an essential component of the regulation of numerous physiological mechanisms including apoptosis [30], aging [31], and myogenesis [32]. As far back as the early 1950s, Warburg demonstrated a link between respiration and proliferation such that cells preparing to proliferate shift to glycolytic metabolism [33]. Multiple studies have demonstrated the involvement of mitochondria in muscle proliferation and differentiation [22–29]. More recently, we have identified that mitochondrial activity is also required for the acute repair of injured muscle fibers through a process that does not depend on mitochondrial ATP production, but instead mitochondrial activity-dependent redox signaling [34, 35]. Further, we have found defects in this specific mitochondrial activity contribute to the poor repair of injured muscle fibers in Duchenne muscular dystrophy [36], myositis [37], and muscular dystrophy due to the lack of MIUC1 [38]. With such a variety of roles, mitochondria are central regulators of the mechanisms involved in muscle growth, repair, and maintenance of mass. In this study, we have demonstrated that CAPN3 deficiency in the skeletal muscle is characterized by mitochondrial dysfunction, which impacts both myoblasts and myofibers. This dysfunction compromises muscle fiber repair, but drugs that promote mitochondrial biogenesis improve myofiber repair in *Capn3*-deficient mice and offer potential new therapies for LGMD2A.

## Materials and methods

### Animal experiments

**Fig. 1:**
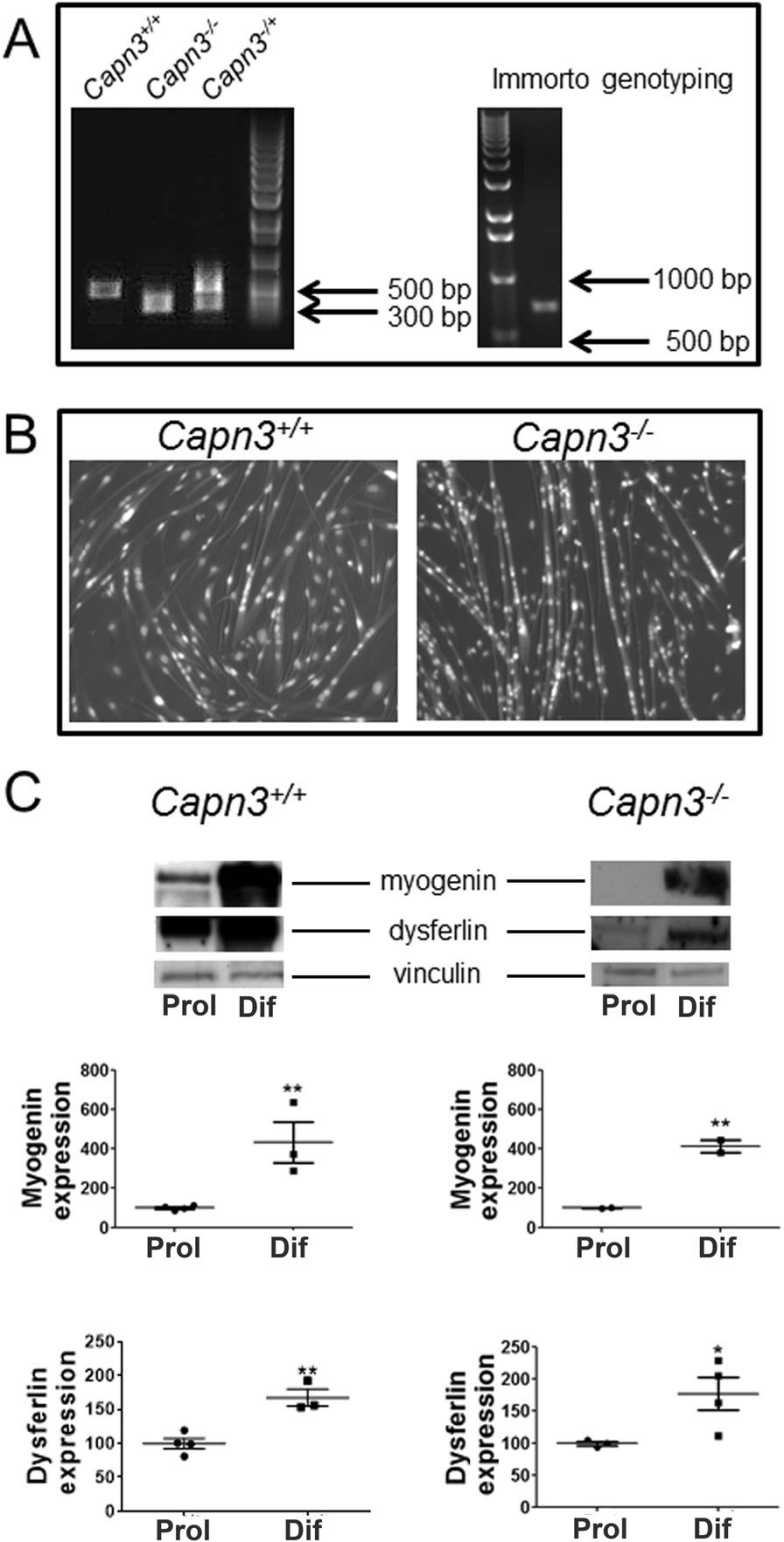
*Capn3*-deficient and *Capn3*-sufficient immortalized primary muscle cells. Genotyping: agarose gel showing the WT (*Capn3*^+/+^) *Capn3* gene band at 500 bp and the *Capn3*-deficient (*Capn3*^−/−^) gene band at 300 bp. The large T gene is shown at 700 bp (**a**). Differentiation test performed on *Capn3*^+/+^ and *Capn3*^−/−^ primary immortalized cell cultures. Hoechst 33342 was used to stain the nuclei and show multinucleated myotubes formation after 2 days in a low-serum medium (**b**). Western blots and quantification of muscle-specific proteins in myoblasts and myotubes. Myogenin and dysferlin were used to verify the myogenicity of cells. The expression of myogenin and dysferlin was normalized to vinculin loading control. Since myogenin and dysferlin expression varied between calpain-deficient and calpain-sufficient myoblasts, we have expressed increased expression of these differentiation markers in myotubes as a percentage of expression seen in myoblasts (**c**). Analyses were performed on *n* = 4 different cell culture flasks for proliferation and *n* = 3 for differentiation. **Significantly different with *p* < 0.01; *significantly different with *p* < 0.05

All mice were handled according to our Institutional Animal Care and Use Committee guidelines under approved protocols. *Capn3*-deficient mice were kindly provided by Dr. Melissa Spencer (the University of California, Los Angeles). *Capn3*-deficient mice were provided on a C57BL/6 background and bred homozygous. Therefore, C57BL/6J mice (The Jackson Laboratory, Bar Harbor, ME) were selected as appropriate controls. All mice were age-matched for experiments. Mice were housed in an individually vented cage system on a 12-h light-dark cycle, with standard mouse chow and water provided ad libitum. *Capn3*-deficient were bred and genotyped according to the method previously described (Fig. 1a) [19]. The 9-month time point chosen for the drug study was based on systematic phenotyping performed in our laboratory on *Capn3*-deficient mice.

### Generation of immortalized *Capn3*-deficient cells

*Capn3*-deficient mice were bred with *H-2K*^*b*^*-tsA58* immortomice (Tg(H2-K1-tsA58)6Kio/LicrmJ, The Jackson laboratory). The F1 generation heterozygotes for *SV40 large T* antigen and the *Capn3*-mutated gene were bred together to produce homozygous mice with the *Capn3*-mutated gene. The following primer pairs were used for genotyping (LTR2: AAA TGG CGT TAC TTA AGC TAG CTT GC; C3upA: GAA AGG GAC AGG AGA AAT GGA G; C3dnB: CCT GAA ACT TCA AGC CTC TGT TC) and *SV40 large T* carrier (forward primer: T ant-R 5′ GAG TTT CAT CCT GAT AAA GGA GG; reverse primer: T ant-F 5′ GTG GTG TAA ATA GCA AAG CAA GC). Three-week-old mice were euthanized, shaved, and placed in iodide water (1/20 v/v) for 5 min, and their extensor digitorum longus (EDL) muscles were harvested from tendon to tendon and incubated in a solution of DMEM + fresh collagenase (2 mg/ml) at 35 °C under agitation for 2 h. After the collagenase digestion, the muscle fibers were isolated with Pasteur pipettes and rinsed in four successive baths of DMEM + 5% penicillin/streptomycin (P/S). Single fibers were plated at 33 °C in 24-well plates coated with Matrigel (in H2K proliferation medium [H2KPM]: 20% heat-inactivated FBS, 4 mM l-glutamine, 2% v/v chick embryo extract, and 1% penicillin/streptomycin [100 U/ml to 100 μg/ml] in DMEM and supplemented with fresh gamma-interferon at 20 U/ml). Satellite cell migration was followed during the next 8 h. Fibers were removed as soon as a few myoblasts or clones were visible. The clones were allowed to proliferate to 70% confluence and harvested, and then the trypsinized cells were placed in a series of culture dishes of varying sizes. The cells were then harvested; one portion was allowed to differentiate, and the cells were then frozen (in H2KPM + 10% DMSO) and stored at − 80 °C.

### Immortalized muscle cell cultures

Conditionally immortalized control C57BL/6 and *Capn3*-deficient myoblast clones contain a temperature-sensitive immortalizing gene, the tsA58 mutation of the SV40 large T antigen, under the control of the H-2K^b^ inducible promoter [39]. When induced with interferon-gamma (IFN-γ), the tsA58 protein is expressed, allowing the cells to proliferate at 33 °C. Myoblasts were plated and maintained on gelatin-coated dishes (0.01% gelatin) in H2KPM at 33 °C and 10% CO_2_ in a humidified incubator. Cultures were fed every 2–3 days with H2KPM, and fresh IFN-γ (20 U/ml) was added to the culture medium at each feeding. Cells were grown to near confluence, harvested with trypsin/EDTA (0.5/0.2 g/l), and centrifuged at 800*g* for 3 min. The cell pellet was used for DNA, RNA, or protein extraction or resuspended in H2KPM. Under these conditions, the cells doubled every 24–30 h at 33 °C and fused with > 50% efficiency into multinucleated sarcomeric tropomyosin-positive muscle fibers when switched to 37 °C in the absence of IFN-γ.

### Gene expression profiling using Illumina BeadChip microarrays

Total RNA was extracted from the tibialis anterior (TA) muscle using TRIzol Reagent and an RNeasy mini kit (Qiagen, Valencia, CA) from the sections of frozen pellets of proliferating cells or cells that had been allowed to differentiate for 5 days. RNA was stored at − 80 °C until used. The quality of the RNA from each sample was assessed using an Agilent RNA 6000 Nano kit in an Agilent 2100 Bioanalyzer (Agilent Technology, Palo Alto, CA). Biotinylated cRNA was prepared from 250 ng of total RNA using an Illumina Totalprep RNA amplification kit (Ambion, Inc., Austin, TX) according to the manufacturer’s protocol. Hybridization of the samples to a MouseWG-6 v2.0 Expression BeadChip (Illumina, Inc., San Diego, CA), washing, labeling with Cy3-streptavidin, and scanning of the BeadChip were performed according to the manufacturer’s protocol. The Illumina data were extracted using Genome Studio (software provided by Illumina), with no background subtraction, then normalized using the “quantiles” function. The data generated from the Genome Studio were then exported to the Partek Genomics Suite software for further analysis. In Partek, the genes differentially expressed between *Capn3*-deficient and *Capn3*-sufficient cells were filtered by a *p* value with a false discovery rate of ≤ 0.05 and a fold change of ≥ 4. The significant networks (identified by using the “Core Analysis” function) associated with the differentially expressed genes for each of the comparisons were analyzed using the Ingenuity Pathway Analysis program (Ingenuity System Inc., CA).

### Assessment of mitochondrial function using flow cytometry analyses

Mitochondrial content, inner membrane potential (ΔΨ), ROS production, and intracellular calcium were assessed with 10-nonyl acridine orange (NAO, Sigma-Aldrich, St. Louis, MO), 3,3′-dihexyloxacarbocyanine iodide (DiOC6, Invitrogen), 2′,7′-dichlorodihydrofluorescein diacetate (H2DFCDA, Invitrogen), and Fluo4-AM (Invitrogen), respectively, as described [25]. Cells were analyzed on a FACSCalibur (BD Biosciences, San Jose, CA) with BD Cell Quest ProTM 4.0.2.

### Analysis of mitochondrial DNA-to-nuclear DNA ratios

Total DNA was extracted from muscle cells using a DNeasy blood and tissue kit (Qiagen). The mitochondrial DNA (mtDNA) ratio was calculated using real-time quantitative PCR by measuring the threshold cycle ratio (ΔCt) of a mitochondrial-coded gene (ND1, forward 5′-GGA CCT AAG CCC AAT AAC GA-3′, reverse 5′-GCT TCA TTG GCT ACA CCT TG-3′) relative to nuclear DNA (nDNA) using a nuclear-coded gene (beta-globulin, forward 5′-CTT CTG GCT ATG TTT CCC TT-3′, reverse 5′-GTT CTC AGG ATC CAC ATG CA-3′).

### Western blotting and LDH activity

Protein homogenates were extracted from the tibialis anterior (TA) muscle as previously described using RIPA buffer (Invitrogen) [40]. Proteins were separated on 4–12% Nupage Bis/Tris gels. After electrotransfer, the membranes were saturated with 5% non-fat dry milk (1 h, 20 °C) and incubated overnight with primary antibody against transcription factor A, mitochondrial (TFAM; 1:500, Cell Signaling), pyruvate dehydrogenase kinase (PDK1) (1:5000, Santa Cruz), dysferlin (1:500, Dako), myogenin (1:1:500, Dako), or vinculin (1:10,000, Sigma), then with the corresponding secondary antibodies (1:3000–1:5000) for 90 min. Immunoreactivity was determined by chemiluminescence and quantified with Quantity One (Bio-Rad).

Lactate dehydrogenase (LDH) activity of muscle lysates was measured using 2.5 μl of protein extract (1:2 dilution) and 225 μl assay buffer (2.5 ml of 1 M Tris [pH 7.6], 500 μl of 200 mM EDTA, 500 μl of 5 mM NADH, and 48 ml water). Oxidation of NADH, H^+^ was recorded after pyruvate addition (10 μl, 100 mM). NADH fluorescence was detected by a luminescence/fluorescence analyzer (Mithras LB 940, Berthold Technologies). LDH activity was normalized to the protein concentration.

### Assessment of cell proliferation using CFSE

Immortalized H2K cells (described above) were cultured in H2KPM, trypsinized, and stained with carboxyfluorescein diacetate succinimidyl ester (CFSE) dye (10 μM, 10 min at 37 °C). One million cells were stored in 4% formalin and kept at 4 °C until analysis. The left-over cells were cultured and harvested at several time points to perform the proliferation kinetics. Cytometry analyses were done to analyze the fluorescence lost during proliferation using the FlowJo software (FlowJo, LLC, OR, USA). We used CFSE fluorescent dye to assess proliferation. A decrease in fluorescence occurs as dyes are evenly diluted into daughter cells with subsequent cell divisions measured as distinct peaks during analysis. The “generation 0” peak was set by drawing a gate around the unstimulated peak in the proliferation assay. The “generation 0” peak represents the undivided cells and had a relatively consistent intensity and coefficient of variation (CV) across the samples collected in the proliferation assay. The fluorescence of CFSE was assessed in each harvested cell time point. The proliferation analysis was applied to each sample. The FlowJo software estimated the optimal peak ratio and CV to represent the population according to the characteristics of the “generation 0.” Each peak represents a successive generation of cell division. The proliferation index was automatically calculated by the software according to the fluorescence analyses. The proliferation index represents the total number of divisions by the number of cells that have undergone division. This gives a more comprehensive understanding of how fast cells are growing indicating the number of divisions completed on average per cell that is proliferating and can reflect the kinetics of behavior of different cell genotypes (*Capn3*-deficient and *Capn3*-sufficient) under the same conditions.

### Treatment of *Capn3*-deficient mice with the PPAR-delta agonist, GW501516

At 8 months of age, the mice were given GW501516 (7.5 mg/kg [80 μl]; Alexis Biochemicals/Enzo Life Sciences) by intraperitoneal injection 5 days/week for 4 weeks. DMSO (1/2 v/v in PBS) was used as a vehicle control. The concentration of DMSO was the same in the drug solution and the vehicle control. Rotarod activity assessments were performed pre- and post-treatment.

### Assessment of motor coordination using Rotarod apparatus

Mice were trained on the Rotarod (Ugo Basile, VA, Italy) for 2 days before data were collected. Each acclimatization session consisted of four training sessions, 2 per day, and each session lasted 120 s at a speed of 5 rpm. Each trial consisted of placing the mouse on the rod at 10 rpm for 60 s (the stabilizing period) followed by acceleration from 10 to 40 rpm within the first 25 s until the animal fell from the rod or until 180 s was reached. If the animals fell during the stabilizing period, they were placed back on the rod to complete the session. The total testing time was 240 s (a 60-s stabilization time and 180-s test time). Each trial was performed twice a day (with a 2-h interval between sessions) for 3 consecutive days. The latency to fall (in seconds) was recorded, and all six scores per mouse were averaged and were recorded as latency to fall (in seconds) for each mouse.

### Behavioral activity measurement

The open-field activity was measured using an open-field digiscan apparatus (Omnitech Electronics, Columbus, OH) before and after drug treatment. All mice were acclimatized for 4 days before the actual data collection. The data were collected every 10 min over a 1-h period each day for 4 days. The horizontal distance, total distance, movement number, and movement number were used to assess overall behavioral activity.

### Serum collection and preparation of muscle lysates

Blood was collected by heart puncture immediately after euthanasia and placed into Eppendorf tubes packed on ice. The blood samples were centrifuged at 4 °C for 10 min at 10,000*g*. The serum was then collected, placed in a new Eppendorf tube, and frozen at − 80 °C until used.

Whole frozen muscles were placed in individual Eppendorf tubes packed on ice with 200 μl of RIPA buffer supplemented with proteinase inhibitor cocktail. The muscles were quickly cut into small pieces with sharp scissors and homogenized with a motorized pestle for 1 min, then centrifuged at 10,000*g* for 10 min at 4 °C. The supernatant was collected and stored at − 80 °C. Before storage, the protein concentration was measured using the Bio-Rad modified-Lowry method.

### RNA extraction from muscle tissue for gene expression profiling

RNA was extracted using a miRNeasy Mini Kit (Qiagen). Reverse transcription (RT) was performed with a TaqMan microRNA reverse transcription kit (Life Technologies Co., Applied Biosystems, Carlsbad, CA). mRNA expression was calculated using real-time quantitative PCR by measuring the threshold cycle ratios (ΔCt) using the following primer pairs for *PPARδ* mRNA (5′-CGC ATG AAG CTG GAG TAC GA-3′, 5′-TGG CAC TTG TTG CGG TTC T-3′), *MyoD* mRNA (5′-TCC TCC AGC CTG TAC TGA CC-3′, 5′-CCT TGG CTC AAC TTC TCT G-3′), *Pax7* mRNA (5′-CTG TGT GGA CAG GCT CAC G-3′, 5′-CAT CAA GCC AGG AGA CAGC-3′), and endogenous control *GAPDH* mRNA (5′-CCG TTC AGC TCT GGG ATG AC-3′, 5′-TTC TCA GCA ATG CAT CCT GC-3′).

### Measurement of creatine kinase

Creatine kinase (CK) determination was performed according to the manufacturer’s instructions using a standard spectrophotometric method with an enzyme-coupled assay reagent from Fisher Scientific (CK10). Absorption at 340 nm was measured every minute for 2 min at 37 °C to calculate the enzyme activity. Duplicate measurements were done on each serum sample, and the data were expressed as units per liter for serum samples or units per protein concentration for muscle lysates.

### Measurement of the contraction properties of the EDL and soleus muscles

Mice were anesthetized with 100 mg/kg ketamine and 10 mg/kg xylazine. The EDL and soleus muscles were isolated and placed in Ringer’s solution (137 mM NaCl, 24 mM NaHCO_3_, 11 mM glucose, 5 mM KCl, 2 mM CaCl_2_, 1 mM MgSO_4_, 1 mM NaH_2_PO_4_, and 0.025 mM turbocurarine chloride) maintained at 25 °C and bubbled with 95% O_2_–5% CO_2_. Contractile properties were measured according to Brooks and Faulkner [41], using an in vitro test apparatus (model 305B, Aurora Scientific). A fatigue protocol was performed for both the EDL and soleus muscles: 5-min duration, one contraction every 4 s, 75 contractions in total. The stimulation duration for the EDL muscle was 300 ms and for the soleus muscle 1000 ms. Both muscles were stimulated with a frequency of 100 Hz. We have assessed specific force (kN/m^2^), twitch-to-tetanus ratio, half-relaxation time (HRT) (seconds), and time to peak tension twitch (TTP) (seconds) of the EDL muscle as well as maximal force relative to baseline for the EDL and soleus muscles was calculated.

### Measurement of membrane repair using laser injury

Freshly isolated whole muscle (biceps brachii, soleus, or EDL) or isolated single fibers from the muscles of 3-month-old male mice were used. The whole muscles were used fresh, while isolated fibers were placed on Matrigel-coated dishes in DMEM and allowed to attach for 1–2 days in an incubator at 37 °C with 5% CO_2_. For imaging response to laser injury, the intact muscles or isolated fibers were transferred to a cell imaging medium previously described [42], incubated with 1.5 μg/ml of *N*-(3-triethylammoniumpropyl)-4-(4-(dibutylamino)styryl)yridinium dibromide (FM® 1-43) (Life Technologies, CA) in Tyrode’s buffer and imaged on an inverted Olympus IX81 microscope (Olympus America, PA) custom-equipped with a pulsed laser, AblateTM (Intelligent Imaging Innovations, Inc. Denver, CO), and diode laser of 488 nm (Cobolt, Sweden). Images were acquired using Evolve 512 EMCCD (Photometrics, Tucson, AZ) at 1 Hz. Image acquisition and laser injury were both controlled using Slidebook 5.0 (Intelligent Imaging Innovations, Inc., Denver, CO). To decrease mitochondrial activity in the wild-type (WT) muscles, carbonyl cyanide 3-chlorophenylhydrazone (CCCP; 2 μM) was added to the preparation before laser injury. To increase the mitochondrial activity in *Capn3*-deficient muscle, pyruvate (100 mM) was added to the preparation 30 min before laser injury. DiOC6 dye (1.5 μM) was used to control the increase in mitochondrial activity with pyruvate treatment; in this case, no FM® 1-43 was used in the muscle because of the spectral overlap in the fluorescence emission of the two dyes.

### Statistical analyses

The mean difference between WT and *Capn3*-deficient mice, muscle, or cells was determined by the unpaired *t* test. Kinetics statistics were measured using 2-way ANOVA.

## Results

### Characterization of *Capn3*-deficient and *Capn3*-sufficient immortomouse muscle cells

Immortalized muscle cells were genotyped for *Capn3* and SV40 large T antigen (Fig. 1a). A portion of the cloned cells was allowed to proliferate until 80–90% of confluence and differentiated for 2 days in a differentiation medium. After 2 days of differentiation, multinucleated fibers with twitching were observed in the cultures (Fig. 1b). Myogenin and dysferlin expression confirmed successful switching of the myoblasts from proliferation to differentiation stages of these cell cultures (Fig. 1c). Further, a comparison between the healthy and *Capn3*-deficient cells showed no difference in the expression levels of both these differentiation marker proteins.

### Gene expression analysis of *Capn3*-deficient and *Capn3*-sufficient muscle cells

**Fig. 2:**
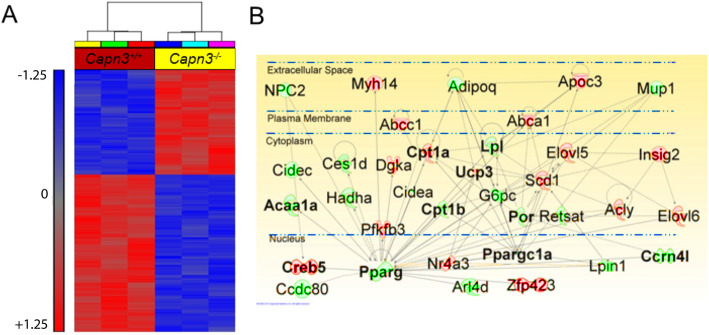
Gene expression profiling of *Capn3*-deficient vs. *Capn3*-sufficient myoblasts with an Illumina BeadChip array. Dendrogram results attesting the good clustering of *Capn3*-deficient (*Capn3*^−/−^) myoblast samples when compared to corresponding WT (*Capn3*^+/+^) muscle cells using Partek Genomics Suite, a statistical analysis and interactive visualization software (**a**). Pathway analysis was done using the Ingenuity Pathways Analysis software that allows functional integration of molecular pathways. Ingenuity pathway analysis demonstrating changes in mitochondrial biogenesis, lipid metabolism, and protein transport in myoblasts. Pink indicates an upregulation and green indicates a downregulation of the specific gene in *Capn3*^−/−^ myoblasts compared to *Capn3*^+/+^ myoblasts (**b**). Three different samples were used per group. cRNA was synthesized from 250 ng of total RNA for each sample. Gene pathways were prepared by ingenuity pathways analysis according to a gene list based on the interaction of a gene candidate with a *p* value of 0.001 and a fold increase ≥ 4

We performed BeadChip arrays to analyze the gene expression patterns in WT and *Capn3*-deficient myoblasts and myotubes. The dendrogram illustrates the hierarchical clustering of differentially expressed genes in WT and *Capn3*-deficient myoblasts (Fig. 2a) and myotubes (Supplemental Fig. 1A). This identified 54 differentially expressed myoblast genes that were directly related to mitochondrial biogenesis, metabolism, lipid metabolism, and protein transport (Fig. 2a, b and Table 1). Comparison of the *Capn3*-deficient myotubes showed differential expression of genes in these same pathways (Supplemental Fig. 1A, B). A list of altered genes related to mitochondrial biogenesis, metabolism, lipid metabolism, and protein transport and their respective fold changes for myoblasts and myotubes is available in the supplemental excel file.

**Table 1:**
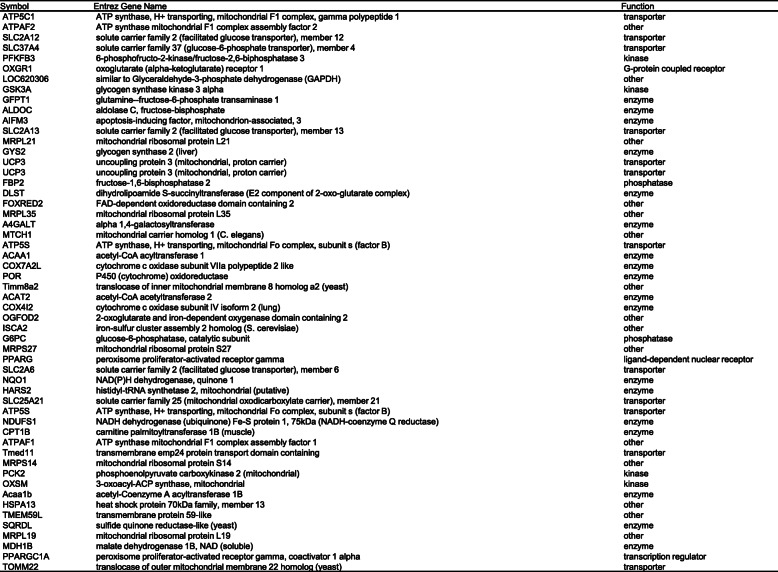
Differentially expressed mitochondrial metabolic genes in *Capn3*^*−/−*^ myoblasts

### Assessment of mitochondrial abundance and activity in *Capn3*-deficient myoblasts

**Fig. 3:**
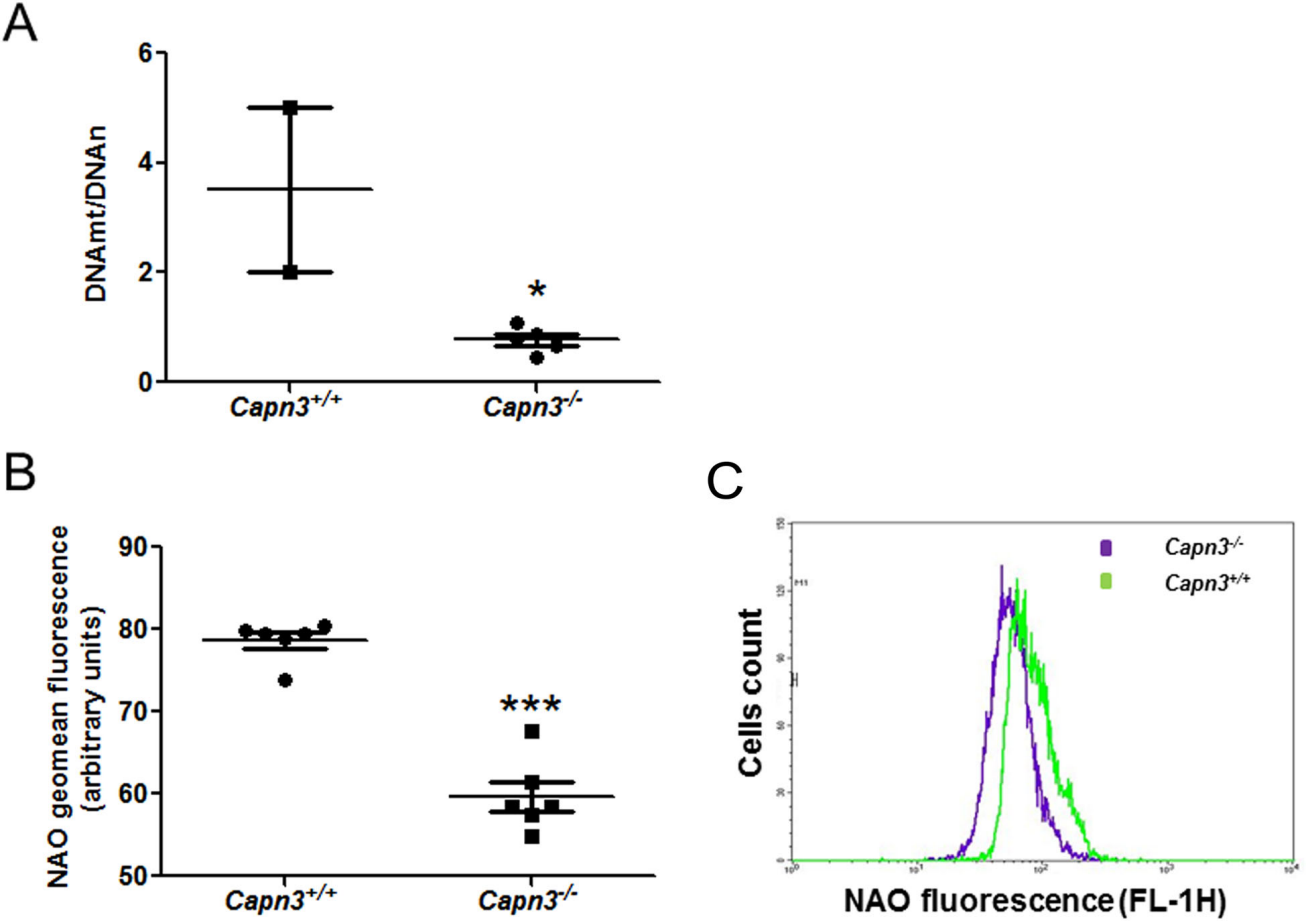
*Capn3* deficiency induces a deficiency in mitochondrial biogenesis in vitro. Analysis of the mitochondrial DNA-to-nuclear DNA ratio in WT (*Capn3*^+/+^) and *Capn3*-deficient (*Capn3*^−/−^) myoblasts. The RT-qPCR analysis was performed on *n* = 2 flasks for *Capn3*^+/+^ and *n* = 6 for *Capn3*^−/−^ (**a**). Flow cytometry analysis of 10-nonyl acridine orange fluorescence (NAO) (cardiolipin content) in WT and *Capn3*^−/−^ myoblasts (**b**, **c**). The analysis was done on *n* = 6 flasks per group. ***Significantly different with *p* < 0.001; *significantly different with *p* < 0.05

With the dysregulation observed in mitochondrial gene expression, we assessed mitochondrial content in myoblasts using the mtDNA/nDNA ratio as a measure for it. This showed that when compared to WT myoblasts, mitochondrial content was reduced by 2-fold in the *Capn3*-deficient myoblasts (Fig. 3a). As another measure of mitochondrial content, we monitored the level of mitochondrial lipid—cardiolipin using the cardiolipin stain 10-nonyl acridine orange fluorescence (NAO). Flow cytometry analysis of NAO fluorescence in live, proliferating myoblasts showed significantly lower fluorescence in *Capn3*-deficient compared to WT cells (Fig. 3b). Together, these results indicate that there is a deficit in mitochondrial content in the *Capn3*-deficient muscle cells.

### Assessment of mitochondrial membrane potential in *Capn3*-deficient myoblasts

**Fig. 4:**
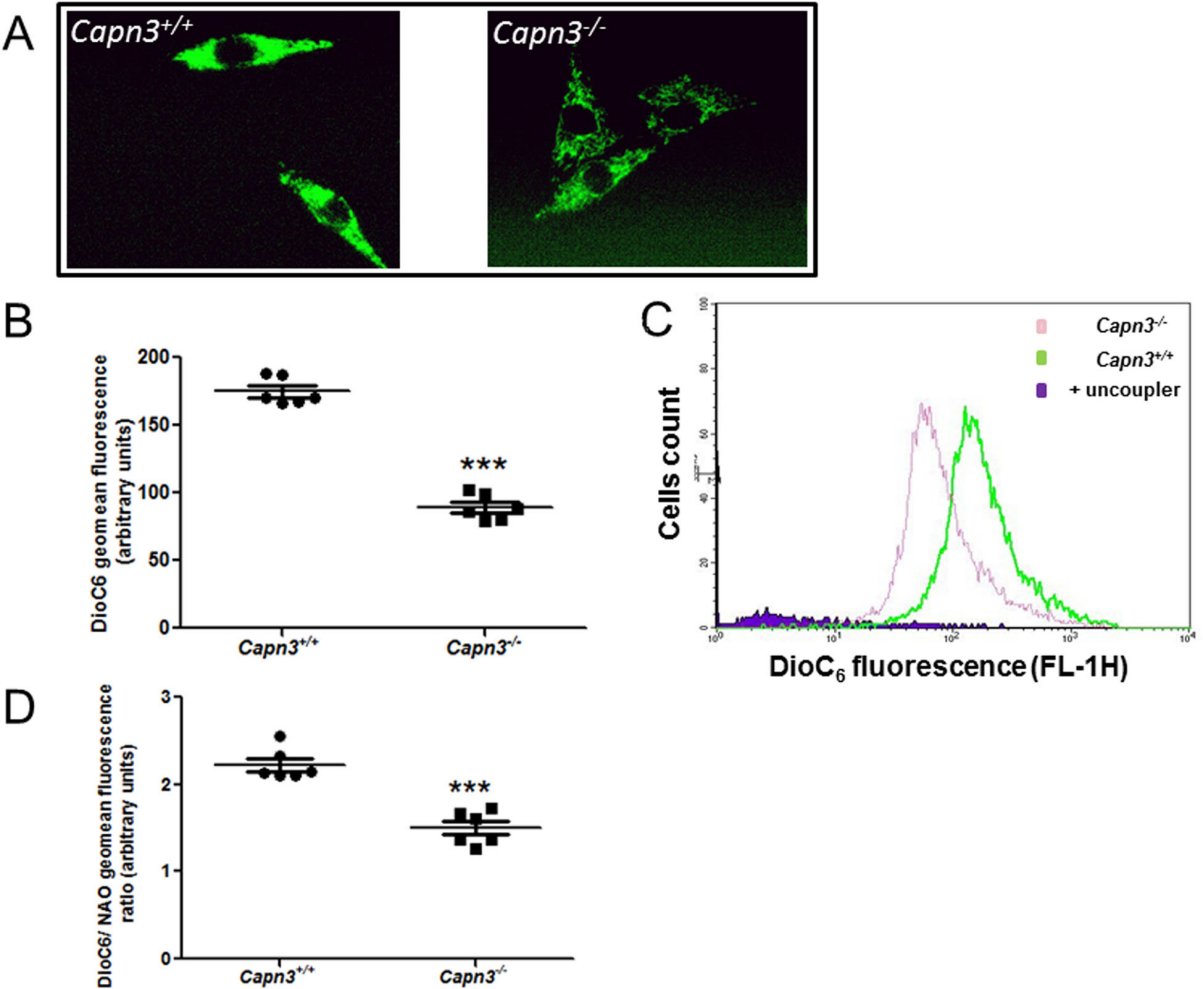
*Capn3* deficiency induces a mitochondrial metabolism deficiency in vitro*.* Live WT (*Capn3*^+/+^) and *Capn3*-deficient (*Capn3*^−/−^) myoblasts stained with DiOC6 to show their mitochondrial inner membrane potentials. Pictures were taken with the same focus, laser intensity, and exposure time in both panels (**a**). Flow cytometric analysis of 3,3′-dihexyloxacarbocyanine iodide (DiOC6) (mitochondrial inner membrane potential) in *Capn3*^+/+^ and *Capn3*^−/−^ myoblasts (**b**). Histogram representing *Capn3*^−/−^ (pink), *Capn3*^+/+^ (green), and *Capn3*^+/+^ and CCCP (uncoupler, purple) DiOC6 fluorescence (**c**). The DiOC6/NAO fluorescence ratio in *Capn3*^+/+^ and *Capn3*^−/−^ myoblasts (**d**). The analysis was done on *n* = 6 flasks per group. ***Significantly different with *p* < 0.001

Next, to assess mitochondrial activity, we monitored the mitochondrial membrane potential (ΔΨ). For this, we measured DiOC6 fluorescence by fluorescent microscopy. WT myoblasts had stronger and denser fluorescent staining than *Capn3*-deficient myoblasts (Fig. 4a). This suggested that *Capn3*-deficient myoblasts have reduced mitochondrial respiration compared to WT cells. When quantitated, *Capn3*-deficient myoblasts had a 2-fold reduced DiOC6 staining (Fig. 4b). The specificity of the DiOC6 staining for mitochondrial inner membrane potential was confirmed by the loss of DiOC6 fluorescence by the mitochondrial uncoupler (CCCP) (Fig. 4c). To assess if reduced DiOC6 labeling reflects reduced mitochondrial abundance or activity, we normalized DiOC6 fluorescence to NAO fluorescence. This normalized fluorescence was also significantly reduced in the *Capn3*-deficient myoblasts, demonstrating that these cells exhibit reduced mitochondrial potential indicative of reduced mitochondrial activity, even after adjusting to mitochondrial content (Fig. 4d).

### Assessment of mitochondrial metabolism in *Capn3*-deficient mouse muscle

**Fig. 5:**
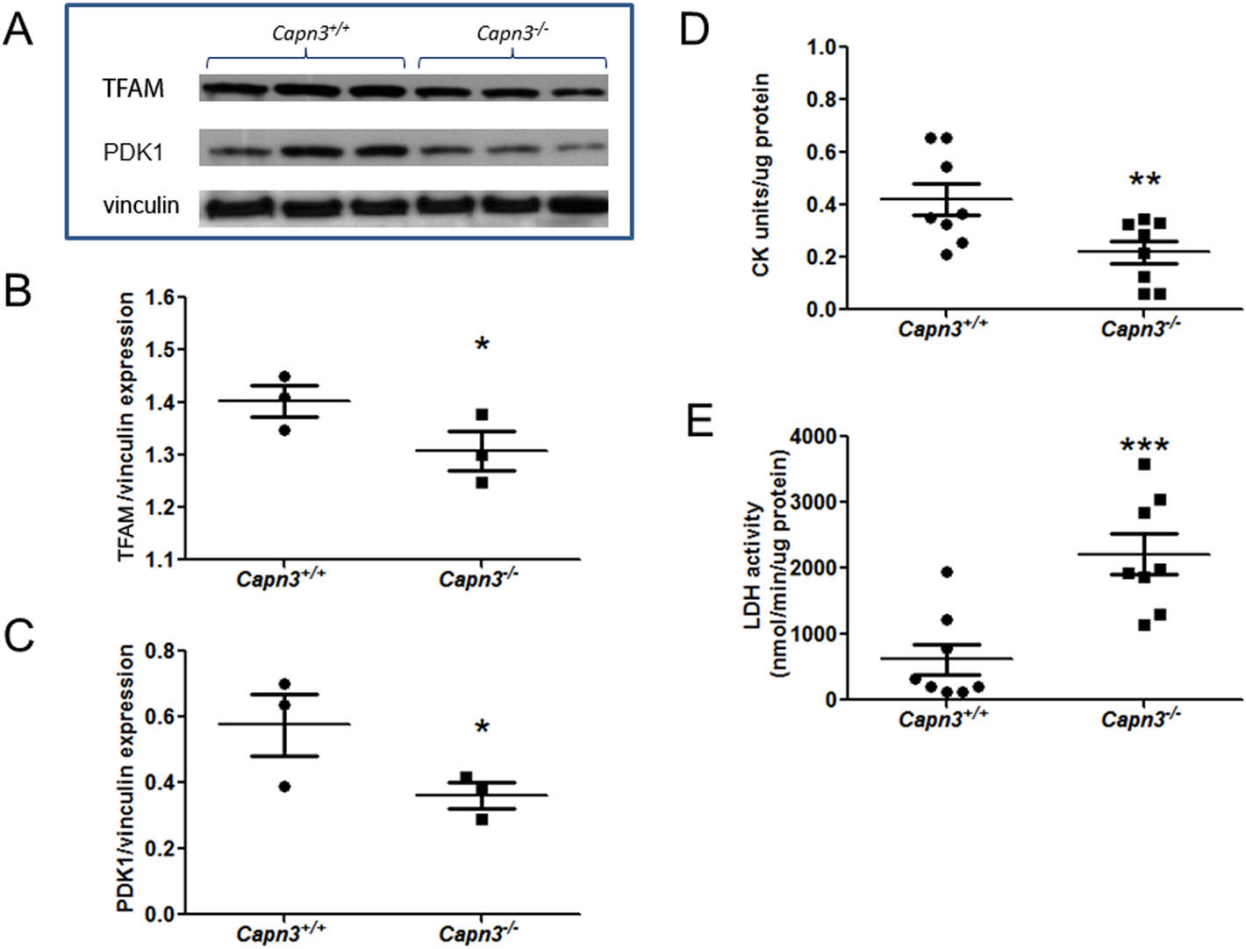
*Capn3* deficiency induces mitochondrial deficiency in vivo*.* Western blot of vinculin (control), TFAM, and PDK1 in the TA muscle lysates from WT (*Capn3*^*+/+*^) and *Capn3*-deficient (*Capn3*^−/−^) mice (**a**). Western blot quantification of TFAM expression in *Capn3*^+/+^ and *Capn3*^−/−^ muscle, normalized to vinculin expression (*n* = 3) (**b**). Western blot quantification of PDK1 expression in the *Capn3*^+/+^ and *Capn3*^−/−^ muscle, normalized to vinculin expression (*n* = 3) (**c**). Creatine kinase (CK) activity in the *Capn3*^+/+^ and *Capn3*^−/−^ TA muscle, normalized to protein concentration (*n* = 8) (**d**). LDH activity measured in the *Capn3*^+/+^ and *Capn3*^−/−^ TA muscle, normalized to protein concentration (*n* = 8) (**e**). ***Significantly different with *p* < 0.001; **significantly different with *p* < 0.01; *significantly different with *p* < 0.05

To examine if the mitochondrial deficit we observed in cultured myoblasts truly represents in vivo mitochondrial deficit in *Capn3*-deficient muscle, we evaluated this deficit in the TA muscles. With the importance of the mitochondrial transcription factor TFAM in mitochondrial biogenesis, we measured the level of this protein and found it to be significantly reduced in *Capn3*-deficient TA muscle compared to WT (Fig. 5a, b). Similarly, using the mitochondrial matrix protein, PDK1, we also found reduced mitochondrial abundance in *Capn3*-deficient tibialis anterior (TA) muscle (Fig. 5a, c). Next, we examined the abundance of the enzyme—creatine kinase (CK) found in the cytoplasm and mitochondria, by measuring the activity of this enzyme in the TA muscle. Compared to WT muscle, CK activity is significantly reduced in the *Capn3*-deficient muscle (Fig. 5d). Concomitantly, we observed that the glycolytic activity (as indicated by LDH activity) was significantly increased in the *Capn3*-deficient muscles as compared to the WT muscles (Fig. 5e). Together, the above findings indicate that mitochondrial biogenesis and activity are compromised both in vivo and in vitro, and the *Capn3*-deficient muscle may be compensating for reduced mitochondrial activity by increased aerobic glycolytic activity.

### Effect of *Capn3* deficiency on myoblast proliferation

**Fig. 6:**
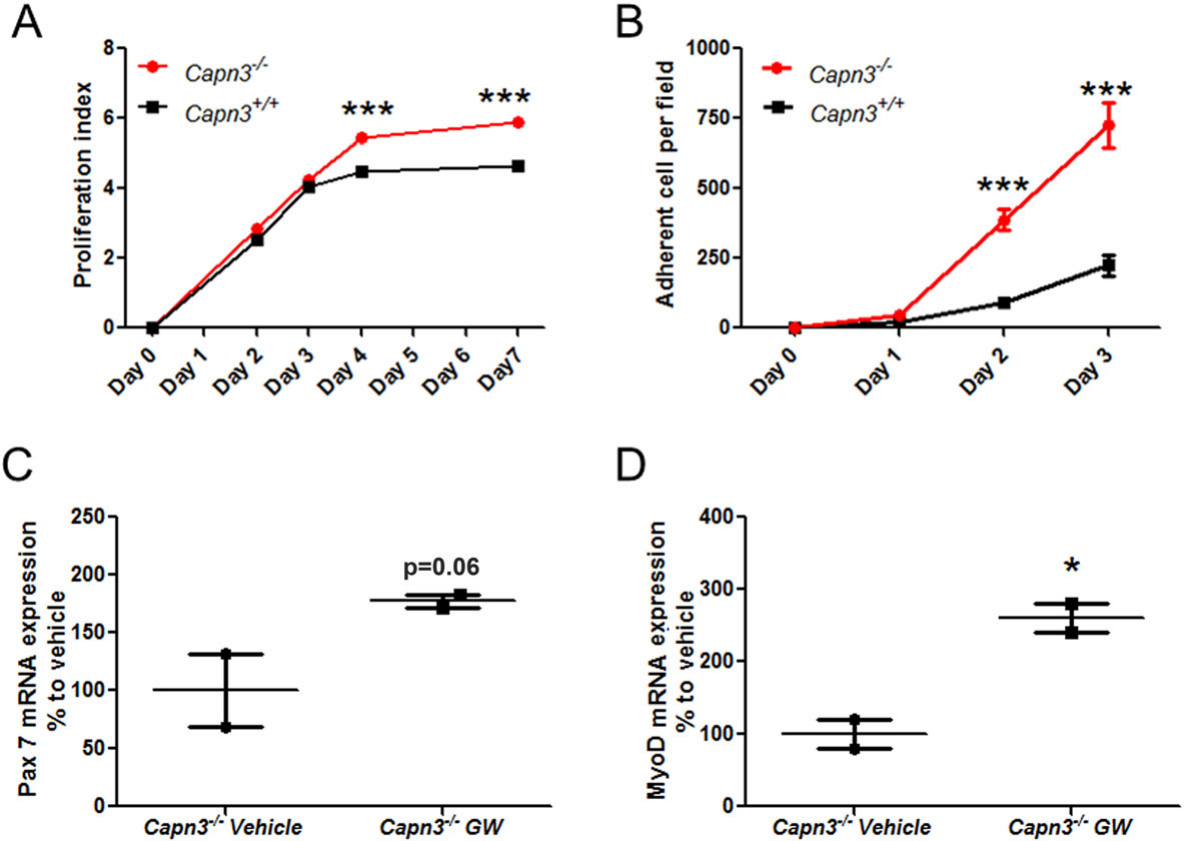
Myoblast proliferation behavior and effect of mitochondrial activity improvement on satellite cell markers. Proliferation kinetics of WT (*Capn3*^+/+^) and *Capn3*-deficient (*Capn3*^−/−^) myoblasts analyzed through flow cytometry by either CFSE fluorescence decrease (*n* = 4) (**a**) or manual counting (*n* = 5) (**b**). Expression of Pax7 mRNA (**c**) and MyoD mRNA (**d**) after 4 weeks of GW501516 treatment in the quadriceps muscle from 7-month-old *Capn3*^−/−^ mice (*n* = 2). ***Significantly different with *p* < 0.001; *significantly different with *p* < 0.05

Since enhanced aerobic glycolysis is associated with increased cell proliferation [33], we examined if the reduced mitochondrial activity and seemingly greater aerobic glycolysis in *Capn3*-deficient cells lead to increased proliferation. To monitor the rate and extent of cell proliferation for *Capn3*-deficient and WT myoblasts, we made use of the CFSE dilution approach (Fig. 6a) as well as cell counting (Fig. 6b). Both these approaches showed that the extent of proliferation of *Capn3*-deficient myoblasts was higher than the WT myoblasts (Fig. 6a, b). This observation shows that in vitro, *Capn3*-deficient myoblasts are more reliant on aerobic glycolysis for their energy provision, which may account for their enhanced ability to proliferate.

### Effect of the PPAR-delta agonist, GW501516, on *Capn3*-deficient mice

In view of the mitochondrial deficit and its consequences on *Capn3*-deficient muscle, we examined if improving mitochondrial biogenesis could be beneficial. We treated *Capn3*-deficient mice with the PPAR-delta agonist, GW501516, which we have previously used to enhance mitochondrial biogenesis in the mdx mouse model [43]. First, we assessed the effect of this treatment on satellite cell activity in vivo by monitoring the expression of *Pax7* and *MyoD* mRNA. We observed an increased expression of *Pax7* (80%) and *MyoD* (160%) mRNA in GW501516-treated *Capn3*-deficient mice (Fig. 6c, d), suggesting that increases in mitochondrial biogenesis increased the muscle satellite cell pool in vivo.

### Effect of the PPAR-delta agonist GW501516 on muscle function in *Capn3*-deficient mice

**Fig. 7:**
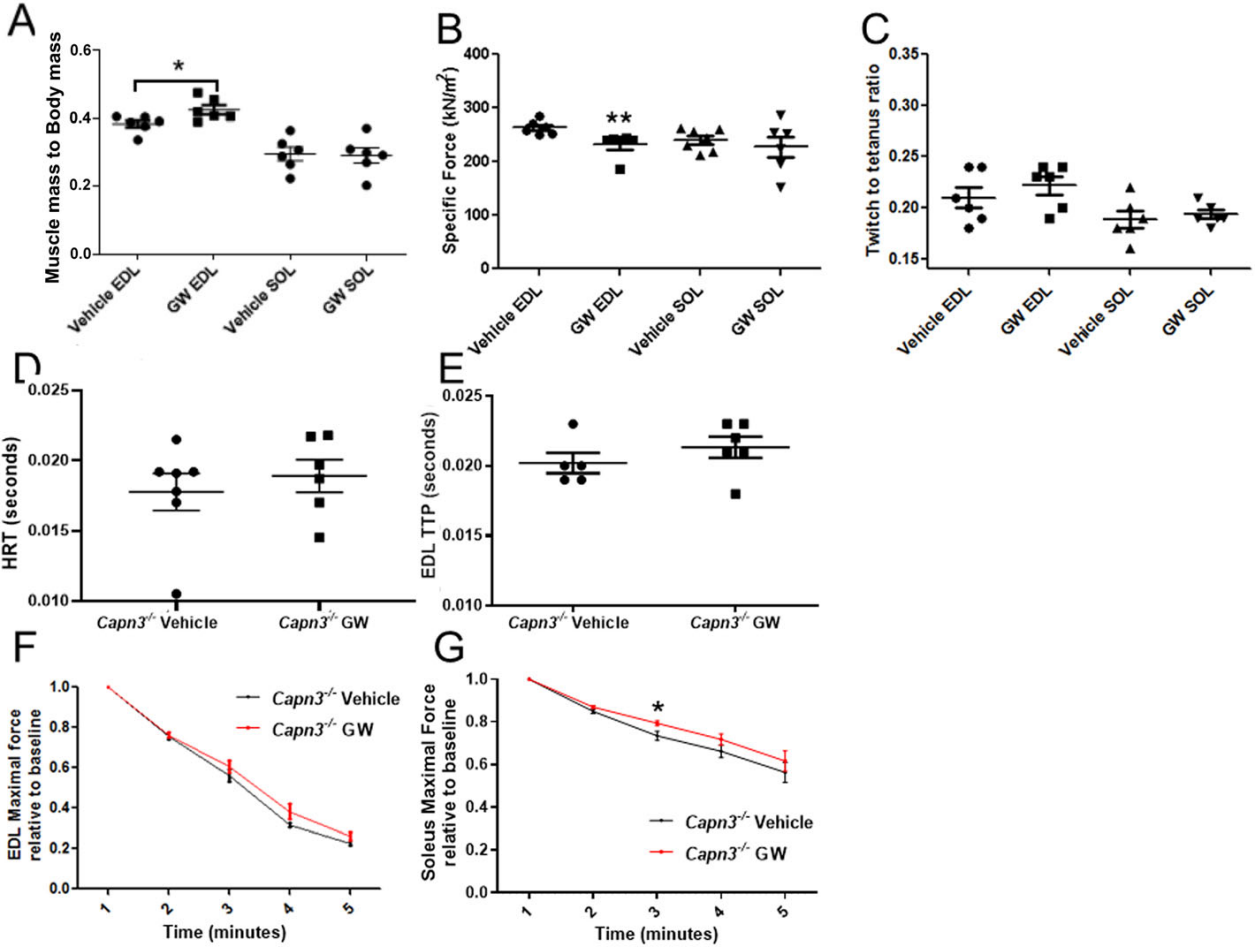
The effect of enhanced mitochondrial activity on muscle phenotype in 9-month-old *Capn3*-deficient mice. EDL and soleus muscle mass (**a**) specific force in *Capn3*-deficient (*Capn3*^−/−^) mice treated with GW501516 (GW) or vehicle (**b**). Twitch-to-tetanus ratio of EDL and SOL in *Capn3*^−/−^ mice treated with GW or vehicle (**c**). Half-relaxation time (HRT) (s) (**d**), time to peak twitch tension (TTP) (s), of EDL muscle (**e**) of GW- or vehicle-treated mice. Maximal force relative to baseline for the EDL (**f**) and soleus (**g**) muscles in *Capn3*^−/−^ mice treated with GW or vehicle. The force is expressed relative to the maximal force at the beginning of the experiment. Significance indicated difference at 3 min

Treatment with PPAR-delta agonist, GW501516, increased the EDL mass relative to body weight but did not change soleus mass relative to body weight (Fig. 7a). Next, we analyzed muscle function, which showed that treatment with GW501516 did not lead to a change in the specific force of the soleus muscle but reduced the EDL-specific force (Fig. 7b). The twitch-to-tetanus force ratios are higher in EDL than in soleus but showed no difference between the vehicle- and drug-treated EDL or soleus muscles (Fig. 7c). We found that GW treatment leads to slower time-to-peak twitch tension (TTP) of the EDL muscle but did not reach statistical significance (Fig. 7d). We did not see differences between the groups for half-relaxation time because of the small sample size and variation (Fig. 7e). EDL maximal force relative to baseline decreased with time but did not show differences between vehicle- and drug-treated mice (Fig. 7f). On the other hand, the soleus muscle showed more fatigue resistance in treated mice compared to vehicle reaching statistical difference at a 3-min time point (Fig. 7 g).

**Fig. 8:**
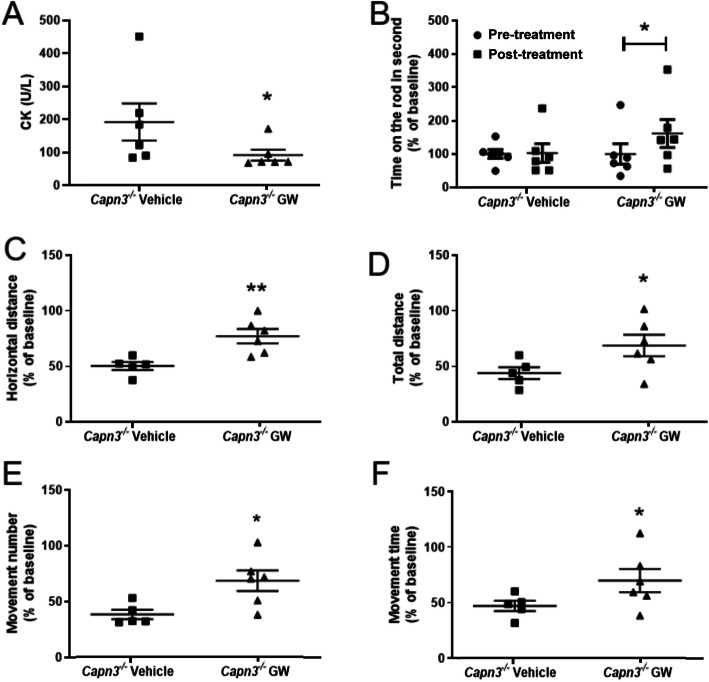
Biochemical and behavioral assessments after GW501516 treatment. Creatine kinase (CK) activity in the blood serum extracted by heart puncture immediately after euthanasia from *Capn3*^−/−^ mice treated with GW or vehicle (**a**). Time on the Rotarod in seconds before and after treatment of *Capn3*^−/−^ mice with GW or vehicle (**b**). Behavioral activity measurements such as horizontal distance (**c**), total distance (**d**), movement number (**e**), and movement number (**f**) were used to assess the overall behavioral activity. Experiments were done with *n* = 6 mice per group. **Significantly different with *p* < 0.01; *significantly different with *p* < 0.05

We observed that the level of CK in the serum was significantly decreased in the GW501516-treated mice (Fig. 8a), suggesting a decrease in muscle leakage and damage. Drug-treated mice also showed a significant improvement in the Rotarod activity (Fig. 8b), suggesting that these mice exhibit better movement coordination after treatment. The open-field activity measurement demonstrated an overall increase in parameters such as horizontal distance (Fig. 8c), total distance (Fig. 8d), movement number (Fig. 8e), and movement time (Fig. 8f), GW501516-treated mice suggesting increase in activity after treatment.

### Effect of muscle injury-induced activation of mitochondrial activity in *Capn3*-deficient muscle

**Fig. 9:**
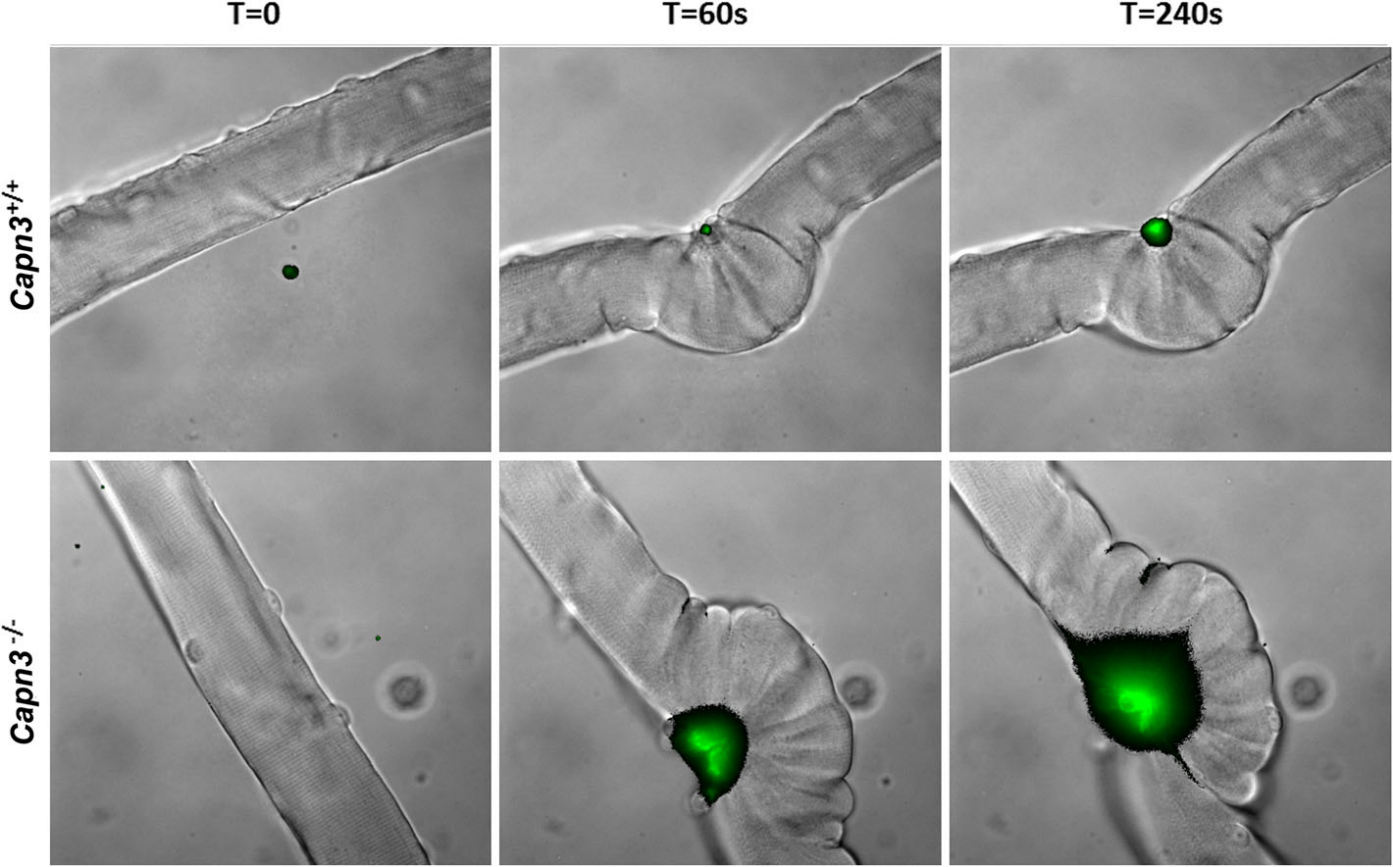
Membrane repair deficits in *Capn3*-deficient muscle after laser injury. Response to focal laser injury in isolated EDL fibers from WT ((*Capn3*^+/+^) top panels at 0, 60, and 240 s) or *Capn3*-deficient ((*Capn3*^−/−^) bottom panels at 0, 60, and 240 s) at 3 months of age. Overlay of the FM 1-43 dye fluorescence (green) on the bright-field (greyscale) image is shown

Reduced mitochondrial and calpain activity compromises muscle cell membrane repair [34, 35, 44–46]. Our findings that *Capn3*-deficient mice show mitochondrial dysfunction and treatment with GW501516 decreases serum CK levels suggest that mitochondrial deficit may underlie poor myofiber repair and addressing this deficit improves myofiber membrane repair. Thus, we evaluated the membrane repair capacity and its dependence on mitochondria activity in the *Capn3*-deficient muscle cells. Response to focal laser injury in isolated EDL fibers from Capn3-deficient mice (Fig. 9, bottom panels at 0, 60, and 240 s) at 3 months of age showed increased dye compared to EDL fibers from WT mice (Fig. 9, top panels at 0, 60, and 240 s).

**Fig. 10:**
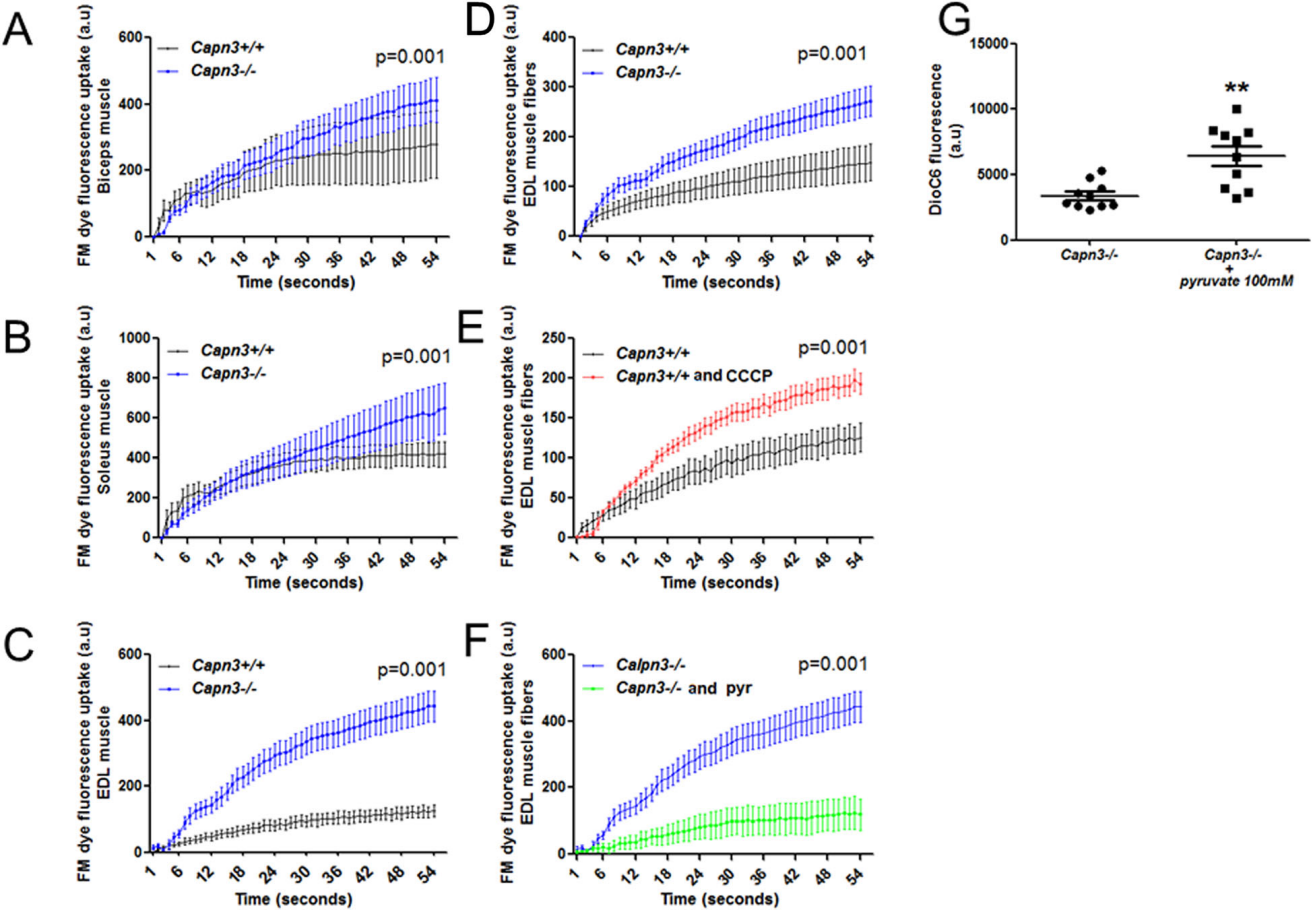
Mitochondrial dependence of membrane repair efficiency after laser injury. Response to laser injury in *Capn3-*deficient (*Capn3*^−/−^) (blue) or WT (black) 3-month-old mice: biceps brachii (*n* = 10) (**a**), soleus (*n* = 10) (**b**), and EDL (*n* = 10) muscles (**c**) and EDL isolated fibers (*n* = 10) (**d**). Effect of using CCCP to uncouple the mitochondrial inner potential in response to laser injury in WT EDL muscle (red) and untreated EDL muscle (black) (*n* = 4 *Capn3*^*+/+*^, *n* = 5 *Capn3*^*+/+*^ + CCCP) (**e**). Response to laser injury in *Capn3*^−/−^ EDL-isolated fibers after activation of mitochondrial activity with pyruvate (100 mM) (green) (*n* = 11), as compared to untreated *Capn3*^−/−^ EDL isolated fibers (blue) (*n* = 3) (**f**). Inner membrane potential assessed with DiOC6 dye fluorescence in *Capn3*^−/−^ EDL-isolated fibers after pyruvate treatment (*n* = 10), as compared to untreated *Capn3*^−/−^ EDL muscle (*n* = 10) (**g**)

We next evaluated to see if this deficit is also present in other muscle types. Muscle fibers from *Capn3*-deficient mice showed reduced ability to repair from focal laser injury, resulting in increased entry of membrane impermeant dye FM 1-43 in the intact biceps brachii (Fig. 10a), soleus (Fig. 10b), and EDL (Fig. 10c) muscles, as well as in the isolated EDL fibers (Fig. 10d). Next, we confirmed that disruption of mitochondrial membrane potential (ΔΨ) with the protonophore CCCP (4 μM) compromised repair of focally injured WT EDL myofibers as shown previously [34, 35] (Fig. 10e). We next examined if enhancing the mitochondrial activity improves repair of *Capn3*-deficient myofibers. To enhance mitochondrial activity, isolated *Capn3*-deficient EDL myofibers were treated for 30 min with 100 mM pyruvate. This increased mitochondrial membrane potential as monitored by the membrane potential-sensitive dye DiOC6 (Fig. 10 g). This increase in mitochondrial activity resulted in the enhanced ability of the injured *Capn3*-deficient myofibers to repair (Fig. 10f). These results demonstrate poor repair of the *Capn3*-deficient myofibers is due in part to the mitochondrial deficit, and increasing mitochondrial activity enables improved repair of the *Capn3*-deficient myofibers.

## Discussion

In the present study, we demonstrated that the muscles lacking CAPN3 have a deficiency in mitochondrial biogenesis and mitochondrial activity, which results in the poor repair of injured myofiber sarcolemma. We tested a potential therapeutic approach in vivo—an activator of mitochondrial biogenesis (GW501516), which showed benefits that hold the promise of addressing the cellular deficits caused by the lack of CAPN3.

Since culturing primary myoblasts is prone to a significant difference between different primary isolates, and since siRNA knockdown in stable cell lines results in significant variability between individual cells, we generated primary myoblast cells from *Capn3*-deficient mice that stably express SV40 large T antigen under an IFNγ-regulated promoter [23]. Through this approach, we produced stable cultures of *Capn3*-deficient primary myoblasts. This facilitated the reproducible investigation of the effects of *Capn3* deficiency in vitro. In order to study the global gene expression changes in *Capn3* deficiency in an unbiased manner, we utilized a BeadChip array, analyzing the expression of 45,000 genes in *Capn3*-deficient myoblasts and myotubes. Expression profiling studies indicated abnormalities in lipid and mitochondrial metabolism. These findings were validated in vitro by performing physiological analysis of mitochondrial (cardiolipin) content and the mtDNA-to-nDNA ratio in living cells, which were reduced in the absence of CAPN3. These findings indicate that *Capn3* deficiency causes a decrease in mitochondrial mass in the muscle cells. We also observed a reduction in mitochondrial membrane potential in the *Capn3*-deficient muscle cells. Analysis of the ratio between mitochondrial content and mitochondrial activity revealed that the mitochondrial defect was not only due to the defect in biogenesis but also in the mitochondrial activity. These data are in agreement with the results of our gene expression profiling experiments, in which we found more than 50 genes related to mitochondrial biogenesis and mitochondrial metabolism to be downregulated in the *Capn3*-deficient muscle cells. While these differences do not provide an insight into the molecular mechanisms that link lipid and mitochondrial abnormalities to CAPN3 deficiency, we did find that *Capn3*-deficient mice exhibit a decreased expression of TFAM, the mitochondrial transcription factor that activates mitochondrial transcription and participates in mitochondrial genome replication. *Capn3*-deficient muscle also showed reduced PDK1 and creatine kinase levels, while the LDH activity was increased, all of which corroborate the mitochondrial dysfunction. Our findings are also supported by previous analyses that have identified structural and biochemical abnormalities in *Capn3*-deficient skeletal muscle mitochondria, which lead to an energy deficit and enhanced ROS levels in the muscles in vivo [21]. While an injury-induced acute increase in mitochondrial ROS is beneficial for membrane repair, we have shown that chronic high levels of mitochondrial ROS inhibit membrane repair [34]. This could be the basis for the poor sarcolemmal repair we observe in the *Capn3*-deficient myofibers.

To understand how mitochondrial abnormalities in *Capn3*-deficient cells contribute to the disease pathogenesis in LGMD2A, we studied the growth and differentiation of myoblasts. Increased aerobic glycolysis we observed in *Capn3*-deficient cells led to enhanced proliferation of these cells compared to the WT myoblasts. Our observation that myoblast proliferation is enhanced by CAPN3 deficit mirrors previous findings that CAPN3 overexpression promotes the renewal of C2C12 myoblast through inhibition of MyoD [47]. In contrast, *Capn3*-deficient C2C12s have a reduced ability to return to quiescence (the G_0_ phase of the cell cycle) after activation, suggesting the stimulation of the cell cycle when CAPN3 is defective. Increased proliferation index for *Capn3*-deficient muscle cells corroborates the putative link between CAPN3 and the transition from G_0_ (quiescence) to G_1_ (cell cycle entrance) in muscle cells. We have now demonstrated that improving mitochondrial biogenesis in 7-month-old *Capn3*-deficient mice increases their expression of MyoD and Pax7 mRNAs in muscle, suggesting that mitochondrial activity improves satellite cell activation but also stimulates the renewal of these cells in vivo.

PPARδ, a transcription factor that regulates mitochondrial biogenesis, has been shown to participate in the renewal of the satellite cell pool [48]. When PPARδ is shut down in the muscles, even at birth, the animals display no difference in body weight, muscle size, or muscle composition, but later, they develop metabolic syndrome and have fewer satellite cells than their WT littermates. Furthermore, the PPARδ-deficient muscles show impaired recovery from cardiotoxin-induced injury [48]. In addition to these previously reported changes, we also found that improving mitochondrial function in *Capn3*-deficient mice by using a PPARδ agonist increased resistance to fatigue in the soleus muscle, increased performance of mice on the Rotarod and open-field activity, and reduced serum CK levels within 4 weeks of treatment. This treatment also reduced EDL-specific force closer to soleus muscle measurements but did not induce significant benefit for reducing EDL fatigue. These data are consistent with the observations derived from genetic overexpression of PPARδ in mice [49]. PPARδ overexpression is sufficient to induce an oxidative muscle phenotype characterized by increased type I muscle fibers and enhanced fatigue resistance. Because EDL is more of a fast-twitch muscle, our observation of reduced specific force may indicate that it was beginning to take on more characteristics of a slower twitch (type I) muscle. With just a 1-month treatment, it is not surprising that we see some tendency, but not a complete switch towards this phenotype. The reduced serum CK levels found in the *Capn3*-deficient mice suggest that muscle damage in these mice is reduced in response to increased mitochondrial activity. It is pertinent to note that future dose-ranging experiments with an appropriate sample size of Calpain3-deficient and Calpain3sufficient mice would need to provide strong evidence to these preliminary observations.

Previously, we showed that optimal mitochondrial functioning is required for sarcolemmal repair, and defects in this lead to multiple muscle diseases [35–38]. Therefore, we investigated the effect of mitochondrial deficit on sarcolemmal repair in the *Capn3*-deficient mice. Previous studies using freshly isolated flexor digitorum brevis (FDB) myofiber in the *Capn3*-deficient mice found no difference in the rate of FM 1-43 dye entry following focal sarcolemmal injury [50]. However, using intact biceps brachii, soleus, and EDL muscles as well as isolated EDL muscle fibers (adhered to Matrigel substrate) from *Capn3*-deficient mice, we found that a lack of CAPN3 compromised the ability of the injured myofibers to repair a focal injury (Fig. 9). The difference in the two observations may reflect the differential effects of CAPN3 deficit that we have observed in the repair ability of the different muscles (EDL > biceps brachii > soleus). In addition, our observations that the repair deficit is most pronounced when the myofibers are injured in intact tissue in the context of proper extracellular matrix support, rather than in isolated myofibers, may be a source of difference between in vivo physiological injury to skeletal myofibers vs. experimental as has been examined previously [50]. The poor sarcolemmal repair in *Capn3*-deficient mice that we observed corresponds to the repair deficit we observed in WT EDL myofibers where the mitochondria were depolarized by CCCP treatment. This implicates mitochondrial dysfunction in the poor sarcolemmal repair seen in the *Capn3*-deficient muscle. This explanation is substantiated by our observation that an increase in mitochondrial activity in *Capn3*-deficient EDL muscle produced by the use of pyruvate not only improved the mitochondrial membrane potential (assessed by DiOC6 fluorescence) but also significantly improved the ability of the *Capn3*-deficient myofibers to heal from laser injury. We have previously demonstrated that increased energy production through the tricarboxylic acid (TCA) cycle is not required for mitochondria-mediated cell membrane repair [34]. Thus, pyruvate-induced improvement in *Capn3*-deficient myofiber repair increase in mitochondrial activity may be mediated by improved mitochondrial calcium homeostasis due to the normalization of the mitochondrial membrane potential deficit in the *Capn3*-deficient muscle. Such a mode of action is supported by our recent demonstration that dysregulation of mitochondrial calcium homeostasis in the skeletal muscle compromises myofiber repair [38]. Independent of the direct mode of action, our data offers support to an important role of mitochondria in the *Capn3*-deficient muscle by way of impairing sarcolemmal repair.

Mitochondrial abnormalities have been described in other muscle diseases such as collagen VI-related myopathies [51, 52], muscular dystrophy due to choline kinase beta (CHKB) gene mutations [53], myositis [37], and Duchenne muscular dystrophy [36]. We and others have previously demonstrated that addressing mitochondrial abnormalities in *mdx* mice can reduce muscle pathology and improve muscle function [43, 54–58]. Thus, our observations suggest an overlap in therapies for muscular dystrophies that improve mitochondrial metabolism.

## Conclusion

This study demonstrates that CAPN3 deficiency in the skeletal muscle is associated with abnormal mitochondria biogenesis and activity. We provide evidence that this phenotype is associated with a dysregulated myoblast proliferation. Mitochondrial impairment also reduces the repair ability of the *Capn3*-deficient myofibers. Taken together, our data identify a role for mitochondrial abnormality as a central contributor to the myofiber and muscle repair pathologies observed due to CAPN3 deficit. By using therapeutic approaches to target the mitochondrial deficit in vivo, we offer a proof-of-concept demonstration of novel drug targets and candidates to treat *Capn3* deficiency in the skeletal muscle.

## Supplementary Information

**Additional file 1: Supplemental Figure 1.** Gene expression profiling of *Capn3*-deficient vs. WT myotubes with an Illumina BeadChip array. Dendrogram results attesting the good clustering of *Capn3*-deficient (*Capn3*^−/−^) myotube samples when compared to corresponding WT (*Capn3*^+/+^) muscle cells (A). Ingenuity pathway analysis demonstrating changes in mitochondrial biogenesis, lipid metabolism, and protein transport in myotubes. Pink indicates an upregulation and green indicates a downregulation of the specific genes in *Capn3*^−/−^ myotubes compared to *Capn3*^+/+^ myotubes (B). Three different samples were used per group. cRNA was synthesized from 250 ng of total RNA for each sample. Gene pathways were prepared by ingenuity pathways analysis according to a gene list based on the interaction of a gene candidate with a *p* value of 0.001 and a fold increase ≥ 4.

**Additional file 2:** FFold changes in WT and calpain-3 deficient myoblasts and myotubes.

## Data Availability

The datasets used and/or analyzed during the current study are available from the corresponding author on reasonable request. BeadChip Array data will be deposited in the GEO database.

## Abbreviations

LGMD2A: Limb-girdle muscular dystrophy type 2A
calpain-3: Human gene: *CAPN3;* mouse gene: *Capn3;* protein: CAPN3
H2KPM: H2K proliferation medium
IFN-γ: Interferon-γ
mtDNA: Mitochondrial DNA
nDNA: Nuclear DNA
ΔΨ: Inner membrane potential
NAO: 10-Nonyl acridine orange
DiOC6: 3,3′-Dihexyloxacarbocyanine iodide
H2DFCDA: 2′,7′-Dichlorodihydrofluorescein diacetate
WT: Wild-type
CK: Creatine kinase
FM 1-43: *N*-(3-triethylammoniumpropyl)-4-(4-(dibutylamino)styryl)yridinium dibromide
