# Supplementary figures and images for: Mitochondrial dysfunction and consequences in calpain-3-deficient muscle
Source: Skelet Muscle. 2020 Dec 11;10:37. doi: 10.1186/s13395-020-00254-1 (PMC7730798; doi:10.1186/s13395-020-00254-1)

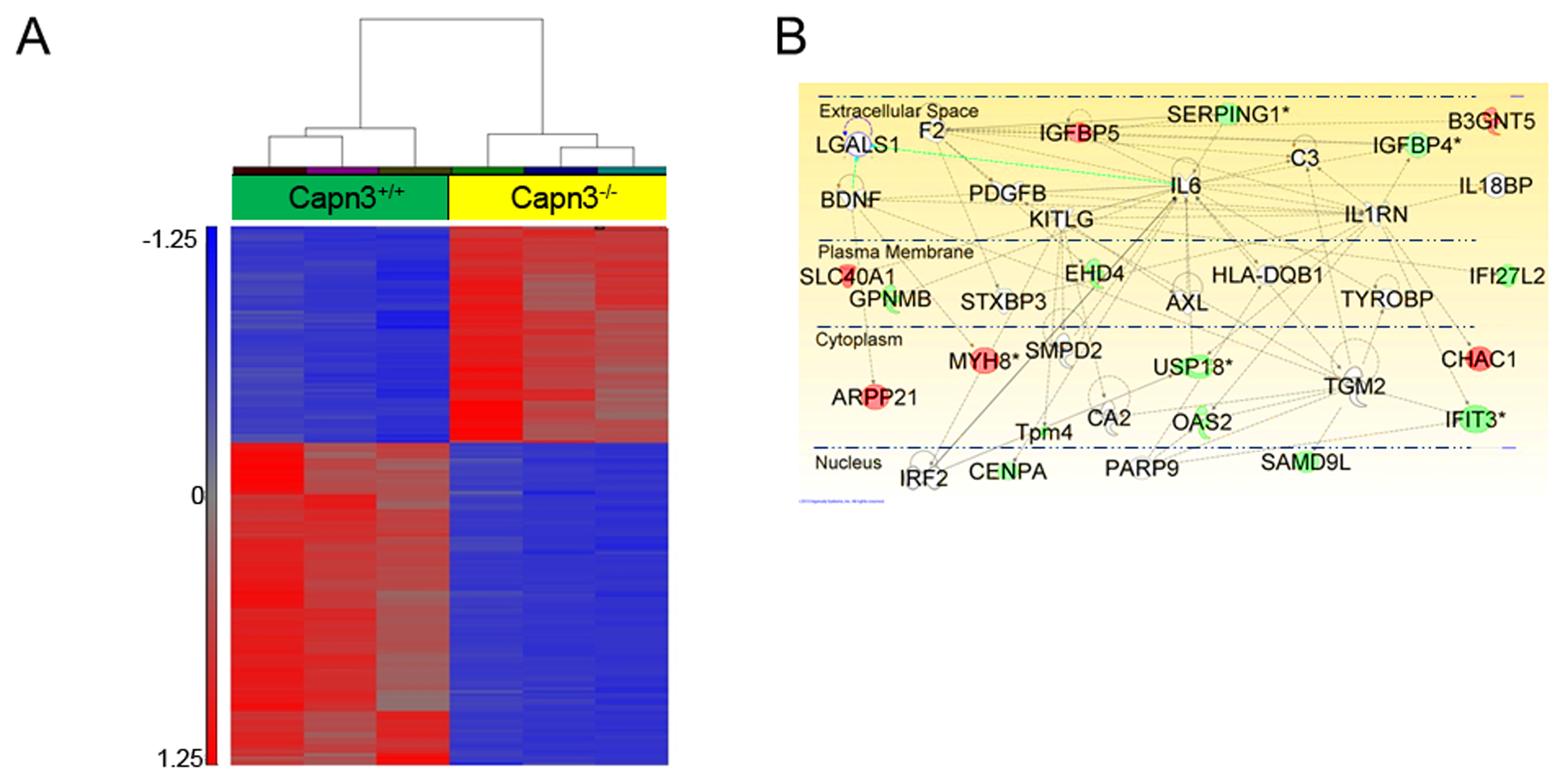

Supplement: Supplementary file 1 — Additional file 1: Supplemental Figure 1. Gene expression profiling of Capn3-deficient vs. WT myotubes with an Illumina BeadChip array. Dendrogram results attesting the good clustering of Capn3-deficient (Capn3−/−) myotube samples when compared to corresponding WT (Capn3+/+) muscle cells (A). Ingenuity pathway analysis demonstrating changes in mitochondrial biogenesis, lipid metabolism, and protein transport in myotubes. Pink indicates an upregulation and green indicates a downregulation of the specific genes in Capn3−/− myotubes compared to Capn3+/+ myotubes (B). Three different samples were used per group. cRNA was synthesized from 250 ng of total RNA for each sample. Gene pathways were prepared by ingenuity pathways analysis according to a gene list based on the interaction of a gene candidate with a p value of 0.001 and a fold increase ≥ 4. [file 13395_2020_254_MOESM1_ESM.tif]
